# Metformin attenuates triglyceride accumulation in HepG2 cells through decreasing stearyl-coenzyme A desaturase 1 expression

**DOI:** 10.1186/s12944-018-0762-0

**Published:** 2018-05-14

**Authors:** Xiaopeng Zhu, Hongmei Yan, Mingfeng Xia, Xinxia Chang, Xi Xu, Liu Wang, Xiaoyang Sun, Yan Lu, Hua Bian, Xiaoying Li, Xin Gao

**Affiliations:** 1 Department of Endocrinology, Zhongshan Hospital, Fudan University, Shanghai, 200032 China; 2 Institute for Metabolic Disease, Fudan University, Shanghai, 200032 China

**Keywords:** Nonalcoholic fatty liver disease, NAFLD, Metformin, Stearyl-coenzyme A desaturase 1, SCD1, Adenosine monophosphate-activated protein kinase, AMPK, Sterol regulatory element-binding protein-1c, SREBP-1c

## Abstract

**Background:**

The prevalence of nonalcoholic fatty liver disease (NAFLD) has increased worldwide. Metformin decreases triglyceride (TG) accumulation in hepatocytes in vivo and in vitro. Stearyl-coenzyme A desaturase 1 (SCD1) knockout mice also show decreased liver TG accumulation; however, whether SCD1 plays a role in the effect of metformin on TG accumulation is unknown. Therefore, the aim of this study was to investigate whether SCD1 mediated the effect of metformin on TG accumulation.

**Methods:**

HepG2 and AML12 cells were exposed to high glucose and high insulin with or without metformin. An adenovirus was used for the SCD1 knockdown and overexpression. The triglyceride level in cells was detected. The expression of related genes was detected by Western blot and quantitative real-time PCR. A dual-luciferase reporter assay was used to determine the effect of metformin on the transcriptional activity of the SCD1 promoter.

**Results:**

Metformin decreased TG accumulation to normal level in HepG2 cells exposed to high glucose and high insulin. The expression of SCD1 and fatty acid synthetase (FAS) was also decreased to normal level by metformin. Knockdown of SCD1 mimicked the effect of metformin on decreasing TG levels in AML12 cells, and the overexpression of SCD1 attenuated the effect of metformin on decreasing TG accumulation in HepG2 cells. The dual-luciferase reporter assay showed that the transcriptional activity of the SCD1 promoter (− 550/+ 199) after metformin treatment was 2-fold lower compared to control group in HepG2 cells. Additionally, the phosphorylation of AMPK after metformin treatment was 2-fold higher, and the expression of sterol regulatory element-binding protein-1c (SREBP-1c) after metformin treatment was about 2-fold lower compared to high glucose and high insulin group in HepG2 cells.

**Conclusions:**

Together, these results reveal that metformin reduces TG accumulation in HepG2 cells via inhibiting the expression of SCD1.

## Background

Nonalcoholic fatty liver disease (NAFLD) is characterized by excessive triglyceride (TG) accumulation in hepatocytes after excluding overt alcohol consumption and other liver injury factors, the spectrum of which ranges from steatosis to nonalcoholic steatohepatitis (NASH) to cirrhosis and even to hepatocellular carcinoma (HCC) [1]. The global prevalence of NAFLD is approximately 25.24% at present [2]. Additionally, the prevalence of NAFLD in China is increasing rapidly, ranging from 6.3% to 27% of the population [3]. NAFLD is considered a hepatic manifestation of metabolic syndrome (MS), and insulin resistance is a key risk factor of NAFLD. A study showed that the prevalence of NAFLD in patients with type 2 diabetes (T2DM) and normal aminotransferase levels reached 36%, which is much higher than that of the general population [4]. In addition, NAFLD is also closely associated with chronic kidney disease, cardiovascular disease, thyroid disease, serum vitamin D level, osteoporosis, serum uric acid level, polycystic ovary syndrome and colon cancer [5–12]. Therefore, there is an urgent need to fight against NAFLD. However, there is no effective drug available for NAFLD at present.

Metformin is a biguanide derivative and is widely used in the treatment of T2DM in clinical practice. Previous studies showed that metformin improved liver fat and serum ALT levels in patients with NAFLD [13, 14], suggesting its potential beneficial effects in the treatment of NAFLD [15]. The probable mechanisms of metformin in the treatment of NAFLD include improvedinsulin resistance, increased fatty acid oxidation, increased glucose uptake in muscle, decreased lipid synthesis, and reduced hepatic glucose production [16]. However, the precise mechanisms underlying the beneficial effects of metformin remain unclear.

Stearyl-coenzyme A desaturase 1 (SCD1) is an enzyme that catalyzes saturated fatty acids to form monounsaturated fatty acids, which is involved in lipid de novo synthesis. SCD1 is related to NAFLD. Studies showed that SCD1 knockout (SCD1 −/−) in mice led to decreased liver TG accumulation, increased fatty acid oxidation, and reduced TG de novo synthesis [17, 18]. In addition, a recent study showed that metformin downregulated SCD1 expression in liver [19]. However, whether SCD1 mediates the beneficial effects of metformin on the treatment of NAFLD is unknown.

Thus, the aim of this study was to explore the role of SCD1 in the effect of metformin on reducing TG accumulation in HepG2 cells. We also investigated whether adenosine monophosphate-activated protein kinase (AMPK)-sterol regulatory element-binding protein-1c (SREBP-1c) pathway was associated with the effect of metformin on reducing SCD1 expression.

## Methods

### Reagents and antibodies

Metformin (#D150959) was purchased from Sigma (St. Louis, USA). The rabbit polyclonal antibodies against SCD1 (#2438), phospho-Thr172 AMPKα (#2535), and AMPKα (#2532) were purchased from Cell Signaling Technology (Cell Signaling, USA).The anti-SREBP-1 antibody (#8984) was purchased from Santa Cruz Biotechnology, Inc. (Santa Cruz, USA). The mouse monoclonal antibody against β-actin was purchased from Sigma (St. Louis). The goat polyclonal secondary antibodies against mouse (#3032) or rabbit (#3012) were both purchased from Signalway Antibody (SAB, USA). The dual-luciferase reporter assay kit was purchased from Promega (Madison, USA).

### Cell culture and treatment

Human HepG2 cells were cultured in DMEM/L media (Gibco, USA) containing 10% fetal bovine serum (Gibco) and 1% penicillin/streptomycin (P/S) (Invitrogen, USA) at37°C and 5% CO2. The immortalized mouse normal hepatocyte AML12 cells were cultured in DMEM/F12 media (Gibco) containing 10% fetal bovine serum, ITS supplement (Gibco), 40 ng/ml dexamethasone, and 1% P/S (Invitrogen) at 37°C and 5% CO2. For the cell experiments, the cells were treated with or without 2 mM metformin in serum-free media containing 30 mM glucose, 100 nM insulin, and 0.25% bovine serum albumin for 24 h.

### Adenovirus preparation and infection

The GV119 vector was used for the construction of adenovirus-shSCD1. The sequences for the shRNA targeting mouse SCD1 and the negative control shRNA were 5’-TTTCTAAGGCTACTGTCTT-3′ and 5’-TTCTCCGAACGTGTCACGT-3′, respectively. The construction, package, amplification, and purification of adenovirus-shSCD1 were performed by the GeneChem Corporation (GeneChem, China). The ADV4 vector was used for the construction of adenovirus-human SCD1. The construction, package, amplification, and purification of adenovirus-human SCD1 were performed by the GenePharma Corporation (GenePharma, China). For the adenovirus infection, the viruses were diluted in PBS and added to media according to the multiplicity of infection (MOI).

### Oil red O stain

The cells were washed with PBS twice and fixed with 4% polyformaldehyde for 15 min. Oil red O stain was performed using Oil red O staining kit (#D027) (Jiancheng Biotech, China) according to the manufacturer’s instructions.

### Cellular TG measurement

The cells in 6-wells plates were washed twice with PBS and digested with 300 μl trypsin for 2 min. Then, 700 μl media was added to stop the digestion. Of the 1000 μl media, 100 μl was used to determine the protein concentration. The remaining 900 μl was used for the TG measurement. Briefly, 900 μl was centrifuged at 800 rpm for 3 min, 1 ml chloroform/methanol (2:1 *v*/v) was added, and the tube was horizontally shaken for 2 h. After that, 500 μl 0.1 M NaCl was added to each tube and mixed well. The mixture was centrifuged at 3700 rpm for 10 min, and the lower layer was transferred to a new tube. The lower layer solution was dried in the chemical hood. After drying, 40 μl 1% Triton X-100-ethol was added to the solution. The concentration of TG was measured via a TG reagent kit (Shensuo UNF, China) according to the manufacturer’s instructions.

### Total RNA isolation and quantitative real-time PCR

The total RNA of the cells was isolated using the TRIzol method according to the manufacturer’s instructions (Takara, Japan). Reverse transcription was performed using an RT reagent kit with gDNA eraser (Takara) according to the manufacturer’s instructions. Quantitative real-time PCR was performed using a SYBR Green Premix Ex Taq (Takara) according to the manufacturer’s instructions. The data were analyzed by the 2^-△△CT^ method. β-actin was used as an internal reference. Table 1 shows the primer sequences of β-actin, SCD1, SREBP-1c, and fatty acid synthetase (FAS).

**Table 1:** Quantitative RT-PCR primers

Gene	Species	Forward primer	Reverse primer
SREBP-1c	human	GCGCCTTGACAGGTGAAGTC	GCCAGGGAAGTCACTGTCTTG
SCD1	human	AGCTCATCGTCTGTGGAGCC	GCCACGTCGGGAATTATGAGG
FAS	human	GGGATGAACCAGACTGCGTG	TCTGCACTTGGTATTCTGGGT
β-actin	human	GATGAGATTGGCATGGCTTT	GTCACCTTCACCGTTCCAGT

### Western blot

A total of 20 μg lysate was loaded onto SDS-PAGE gels and transferred to polyvinylidenedifluoride (PVDF) membranes (Millipore, USA). Then, the membranes were blocked for 60 min with 5% skim milk prepared in TBST (Tris-buffered saline containing 0.1% Tween-20) at room temperature. The membranes were incubated overnight with the primary antibodies diluted in 5% bovine serum albumin prepared in TBST at 4°С. After washing three times for 5 min in TBST, the membranes were incubated with the HRP-conjugated secondary antibody for 1 h at room temperature. After washing three times for 5 min in TBST, the protein bands were detected by Immobilon Western Chemiluminescent HRP Substrate (Millipore) according to the manufacturer’s instructions. The densitometric quantification analysis was performed via ImageJ software (http://imagej.nih.gov/ij/).

### Dual-luciferase reporter assay

Luciferase reporter plasmids encoding the SCD1 promoter between − 920/+ 199 and − 550/+ 199 were gifts from Prof. Yu Li. The HepG2 cells were cotransfected with 0.5 μg of empty vector, along with 0.5 μg of luciferase reporter plasmids and 50 ng of renilla luciferase plasmid (Promega, USA) as an internal control in 12-well plates via FuGENE (Promega) according to the manufacturer’s instructions. After 24 h, the media was changed, and the HepG2 cells were treated with metformin (2 mM) for 24 h in serum-free medium. Dual-luciferase reporter assays were measured and analyzed according to the manufacturer’s instructions (Promega).

### Statistical analysis

All the data were presented as the mean ± standard error of the mean (SEM). One-way ANOVA and post hoc multiple comparisons (LSD method) were performed for intergroup comparisons. A *P* value of less than 0.05 was considered a significant difference.

## Results

### Metformin decreases TG accumulation in HepG2 cells exposed to high glucose and high insulin

**Fig. 1:**
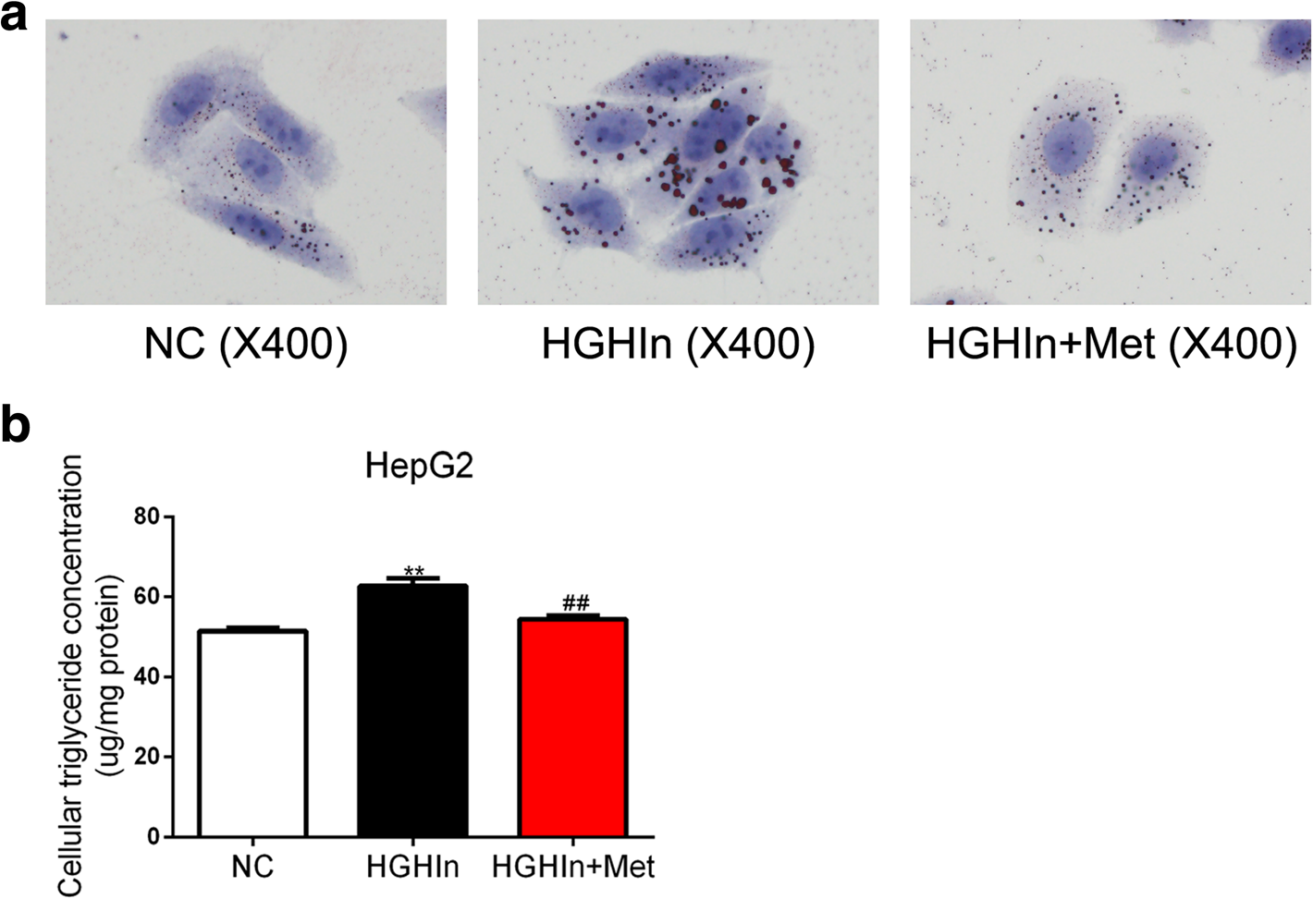
Metformin reduces TG accumulation in HepG2 cells exposed to high glucose and high insulin. **a** Representative gross morphology of Oil red O staining of HepG2 cells. **b** Cellular TG concentration of HepG2 cells. The data are presented as the means ± SEM, *n* = 4. ^**^p < 0.01, versus NC; ^##^*p* < 0.01, versus HGHIn. NC normal control, HGHIn high glucose high insulin; Met metformin

We investigated whether metformin could decrease TG accumulation in HepG2 cells. The HepG2 cells were exposed to high glucose (30 mM) and high insulin (100 nM) with or without metformin (2 mM) for 24 h. Oil red O staining showed that high glucose and high insulin increased TG accumulation in the HepG2 cells, while treatment with metformin substantially decreased TG accumulation in the HepG2 cells (Fig. 1a). Then, we extracted TG from the whole cells for quantitative analysis. As shown in Fig. 1b, TG accumulation induced by high glucose and high insulin was substantially reduced by metformin. These findings reveal that metformin decreases TG accumulation induced by high glucose and high insulin in HepG2 cells.

### Metformin decreases SCD1 expression in HepG2 cells exposed to high glucose and high insulin

**Fig. 2:**
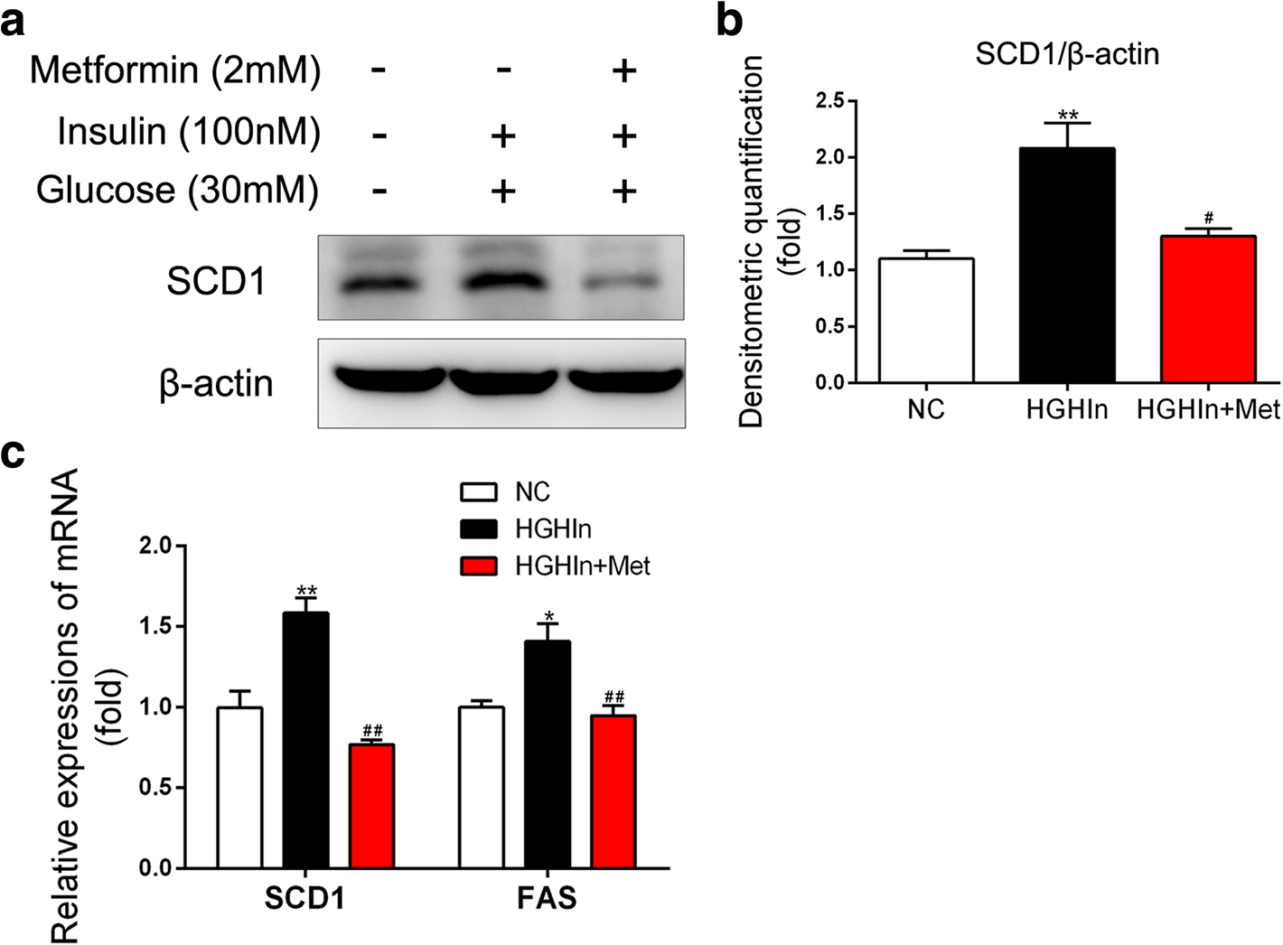
Metformin decreases the expression of lipid de novo synthesis-related genes in HepG2 cells exposed to high glucose and high insulin. **a** and **b** Metformin decreases the protein expression of SCD1 in HepG2 cells exposed to high glucose and high insulin. The data are presented as the means ± SEM, *n* = 3. ^**^p < 0.01, versus NC; ^#^*p* < 0.05, versus HGHIn. **c** Metformin decreases the mRNA expression of FAS and SCD1 in HepG2 cells exposed to high glucose and high insulin. The data are presented as the means ± SEM, n = 4. ^*^p < 0.05 and ^**^p < 0.01, versus NC; ^##^*p* < 0.01, versus HGHIn. NC normal control, HGHIn high glucose high insulin; Met metformin

We next explored the expression of SCD1 after metformin treatment in HepG2 cells. The expression of SCD1 at both the protein and messenger RNA (mRNA) levels was increased when the HepG2 cells were exposed to high glucose and high insulin, and metformin dramatically decreased the expression of SCD1 at both the protein and mRNA levels (Fig. 2a-c). We also explored the expression of other genes involved in TG biosynthesis. The mRNA expression of FAS, a key enzyme in fatty acid synthesis, was further determined by real-time PCR. The results showed that metformin reduced the amounts of FAS, which was increased approximately 1.5-fold in the high glucose and high insulin conditions (Fig. 2c). Taken together, these results suggest that metformin decreases the expression of SCD1 and other triglyceride biosynthesis-related genes in HepG2 cells exposed to high glucose and high insulin.

### SCD1 mediates the effect of metformin on decreasing TG accumulation in HepG2 cells exposed to high glucose and high insulin

**Fig. 3:**
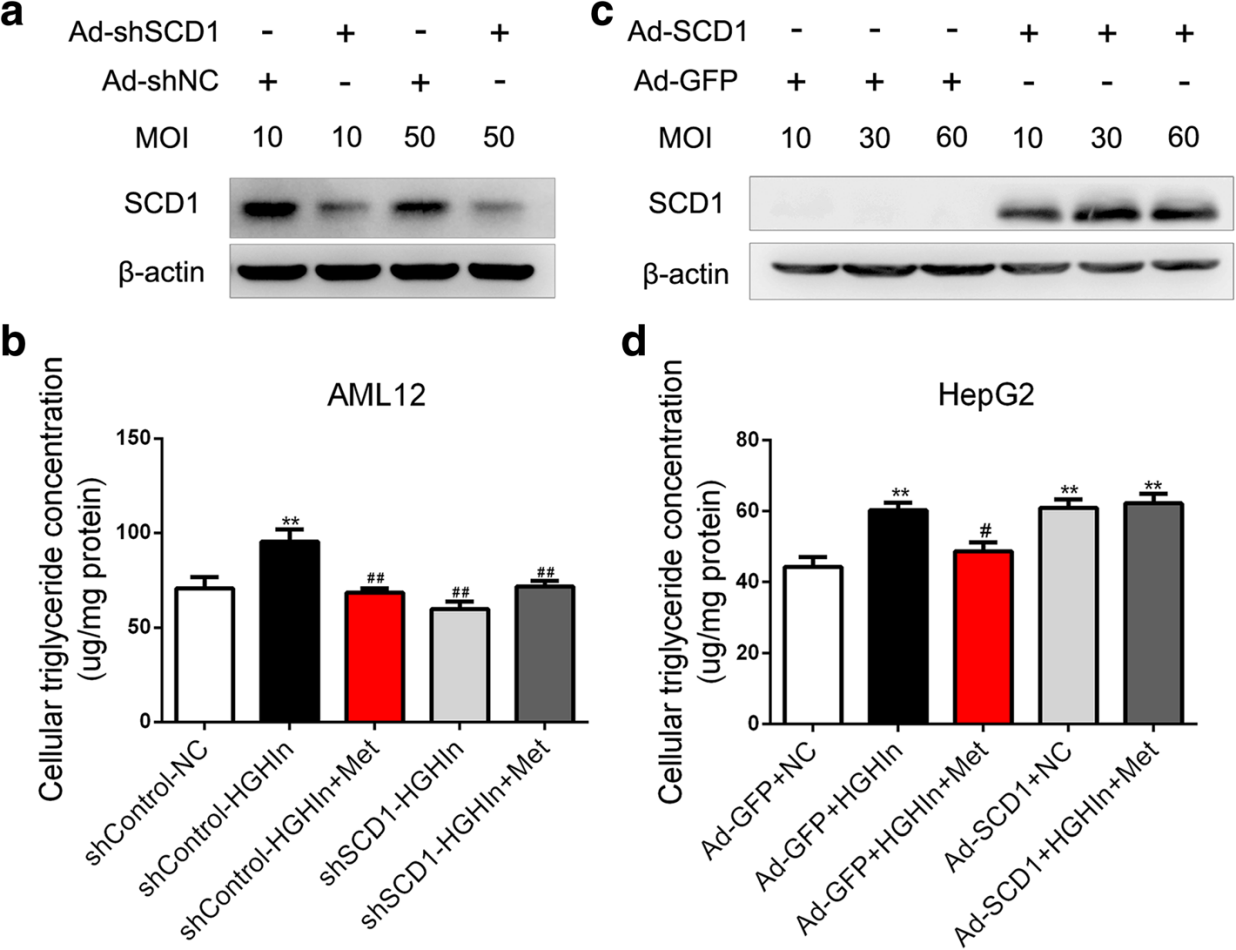
SCD1 is involved in the effect of metformin on decreasing TG accumulation in HepG2 cells exposed to high glucose and high insulin. **a** and **b** Silencing of SCD1 by shRNA in AML12 cells exposed to high glucose and high insulin simulates the effect of metformin on decreasing TG levelin AML12 cells exposed to high glucose and high insulin. The data are presented as the means ± SEM, *n* = 4. ^**^p < 0.01, versus NC; ^##^*p* < 0.01, versus HGHIn. **c** and **d** Overexpression of SCD1 attenuates the effect of metformin on decreasing TG accumulation in HepG2 cells exposed to high glucose and high insulin. The data are presented as the means ± SEM, *n* = 4. ^**^p < 0.01, versus NC; ^#^*p* < 0.05, versus HGHIn. NC normal control, HGHIn high glucose high insulin; Met metformin

To investigate whether SCD1 mediated the effect of metformin on decreasing TG accumulation, we explored whether SCD1 mediated the process of TG accumulation in the cytoplasm. An adenovirus encoding SCD1 small hairpin RNA (shRNA) was used to silence SCD1 in AML12 cells. As Fig. 3a shows, MOIs of both 10 and 50 silenced the expression of SCD1 in the AML12 cells. Therefore, an MOI of 50 was applied for the next experiments. When high glucose and high insulin were added, the TG level in the cytoplasm was increased, and metformin substantially reduced TG accumulation in the AML12 cells (Fig. 3b). Interestingly, silencing SCD1 in high glucose and high insulin conditions reduced TG accumulation in the AML12 cells, simulating the effect of metformin on decreasing TG levels (Fig. 3b). These findings reveal that SCD1 mediates the process of TG accumulation in AML12 cells with high glucose and high insulin.

We next explored whether SCD1 mediated the effect of metformin on decreasing TG accumulation. An adenovirus encoding human *SCD1* was used to overexpress SCD1 in HepG2 cells. MOIs of 10, 30, and 60 overexpressed SCD1 in HepG2 cells (Fig. 3c). Therefore, an MOI of 30 was applied for the next experiments. As Fig. 3d shows, the overexpression of SCD1 increased TG accumulation in HepG2 cells in normal conditions, and the overexpression of SCD1 attenuated the effect of metformin on decreasing TG accumulation. These findings reveal that SCD1 mediates the effect of metformin on decreasing TG accumulation in HepG2 cells.

### Metformin decreases SCD1 expression via inhibiting the transcriptional activity of the SCD1 promoter in HepG2 cells

**Fig. 4:**
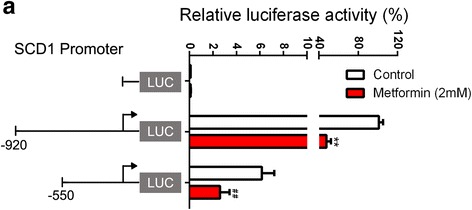
Metformin inhibits the transcriptional activity of the SCD1 promoter in HepG2 cells. **a** The SCD1 promoter between both − 920/+ 199 and − 550/+ 199 is inhibited by metformin, and the element responsible for metformin functionis in − 550/+ 199. The data are presented as the means ± SEM, *n* = 3. ^**^p < 0.01, versus control in SCD1 promoter of − 920/+ 199; ^##^*p* < 0.01, versus control in SCD1 promoter of − 550/+ 199

Because metformin decreased both protein and mRNA expression of SCD1 in the HepG2 cells, we hypothesized that metformin might decrease SCD1 expression via inhibiting the transcriptional activity of the SCD1 promoter. As Fig. 4a shows, the transcriptional activation of different lengths of SCD1 promoters (− 920/+ 199 and − 550/+ 199) was markedly inhibited by metformin in the HepG2 cells, indicating that the element responsible for metformin action was in the SCD1 promoter region (− 550/+ 199). These findings suggest that metformin decreases SCD1 expression via reducing the transcriptional activity of the SCD1 promoter (− 550/+ 199) in HepG2 cells.

### Metformin reduces SCD1 expressionprobably via the AMPK-SREBP-1c pathway

**Fig. 5:**
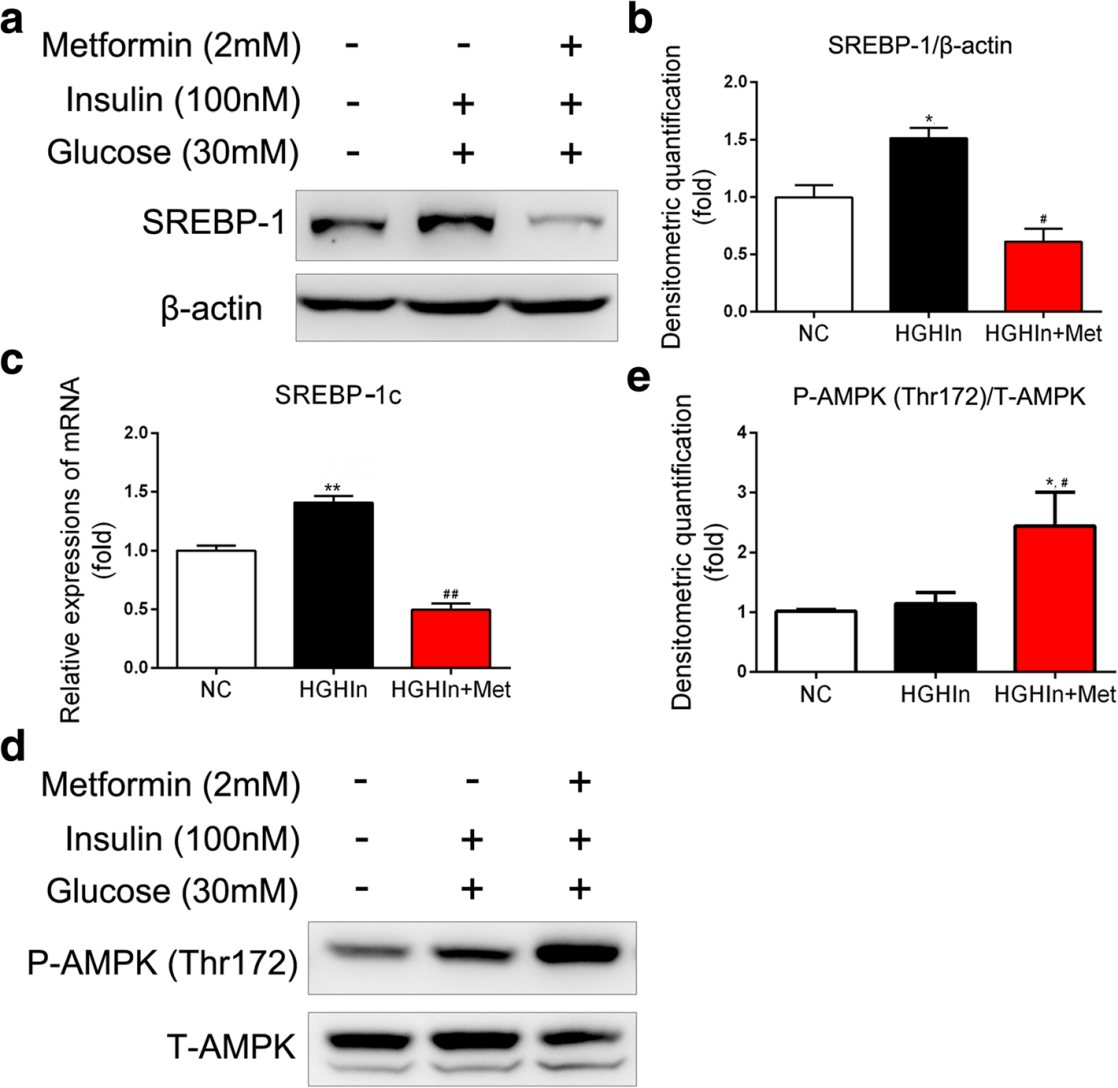
AMPK-SREBP-1c pathway may be associated with the function of metformin in reducing SCD1 expression. **a**-**c** The expression of SREBP-1c is decreased by metformin in HepG2 cells exposed to high glucose and high insulin. The data are presented as the means ± SEM, *n* = 3–4. ^*^p < 0.05 and ^**^p < 0.01, versus NC; ^#^*p* < 0.05 and ^##^*p* < 0.01, versus HGHIn. **d** and **e** The phosphorylation of AMPK is increased by metformin in HepG2 cells exposed to high glucose and high insulin. The data are presented as the means ± SEM, *n* = 3. ^*^p < 0.05, versus NC; ^#^*p* < 0.05, versus HGHIn. NC normal control, HGHIn high glucose high insulin; Met metformin

SREBP-1c, a key transcription factor in the regulation of de novo lipid synthesis in the liver, is considered to be upstream of SCD1 [20]. As Fig. 5a-c shows, the mRNA and protein expression of SREBP-1c increased in the condition of high glucose and high insulin, and the mRNA and protein expression of SREBP-1c was substantially reduced after treatment with metformin. SREBP-1 has two isoforms, SREBP-1c and SREBP-1a. Because SREBP-1a is the less-abundant isoform in the liver, the changes in SREBP-1 protein in hepatocytes mainly represent SREBP-1c, although the antibody used in this study recognizes both isoforms.

A previous study showed that metformin increased the phosphorylation of AMPK, and the phosphorylation of AMPK reduced the expression of SREBP-1c, thus mediating the effect of metformin on decreasing TG accumulation [21]. To verify this finding, we next investigated the phosphorylation of AMPK in HepG2 cells after metformin treatment. As shown in Fig. 5d and e, metformin substantially increased the phosphorylation of AMPK in HepG2 cells.

These data indicate that the AMPK-SREBP-1c pathway may be involved in the effect of metformin on decreasing TG accumulation in HepG2 cells.

## Discussion

Both in vivo and in vitro studies reveal that metformin reduces TG accumulation in hepatocytes and improves NAFLD [13, 14, 22, 23]. However, whether SCD1 plays a role in the effect of metformin on NAFLD is unknown. In this study, we demonstrated that metformin decreased TG accumulation in hepatocytes, the mechanism of which was via decreasing the expression of SCD1 by inhibiting the transcriptional activity of SCD1. Knockdown of SCD1 simulated the effect of metformin on reducing TG level in hepatocytes, and the overexpression of SCD1 attenuated the effect of metformin on reducing TG accumulation in hepatocytes.

Insulin resistance is an important factor in the development of NAFLD. To simulate this environment, high glucose and high insulin conditions were used to build the NAFLD cell model, which was also applied in another study [21].

Previous studies indicate that metformin reduces TG accumulation in hepatocytes through inhibiting TG de novo synthesis [16]. In support of this result, in the present study, we found that metformin decreased TG accumulation in hepatocytes through decreasing the expression of SCD1, which belongs to the TG de novo synthesis pathway. Additionally, the expression of SREBP-1c, a key transcription factor in the regulation of lipid de novo synthesis in the liver, was also decreased by metformin, which is consistent with previous studies [19, 21].

Previous studies showed that SCD1 knockout mice presented decreased TG levels in the liver, and metformin decreased the expression of SCD1 in fatty liver disease [17–19, 21]. However, the precise role of SCD1 in the effects of metformin on NAFLD is unknown. In the present study, we also found that SCD1 was involved in TG accumulation in hepatocytes exposed to high glucose and high insulin. Additionally, we found that metformin decreased the expression of SCD1 at both the mRNA and protein levels, and SCD1 mediated the effects of metformin on TG accumulation in hepatocytes exposed to high glucose and high insulin.

We further explored the potential mechanisms of metformin on decreasing the expression of SCD1. The study showed that SREBP-1c and nuclear receptor TR4 were upstream of SCD1 and positively regulated SCD1 via regulation of SCD1promoter activity [19, 24]. In the present study, although both SREBP-1c and SCD1 were downregulated by metformin, and SCD1 promoter activity was also downregulated by metformin, we did not know whether SREBP-1c mediated the regulation of SCD1 by metformin, which should be explored in the future.

It is reported that metformin is an agonist of AMPK, and AMPK plays a key role in cell metabolism, including lipid de novo synthesis [19, 21, 25, 26]. In the current study, we found that metformin promoted the phosphorylation of AMPK. However, whether AMPK is involved in the regulation of SCD1 by metformin is unknown. Additionally, AMPK is upstream of SREBP-1c and can directly phosphorylate SREBP-1c (Ser372) [21]. Metformin increases the phosphorylation of AMPK, and the phosphorylation of AMPK promotes the phosphorylation of SREBP-1c [21]. The phosphorylation of SREBP-1c inhibits the nuclear translocation of SREBP-1c, thus leading to reduced lipid de novo synthesis [21]. In the current study, although the phosphorylation of AMPK was increased and the expression of SREBP-1c was reduced after metformin treatment, we did not detect the phosphorylation of SREBP-1c (Ser372). Therefore, whether the AMPK-SREBP-1c pathway plays a role in the regulation of SCD1 by metformin is uncertain.

There are some limitations in this study. First, these results were obtained from cell experiments, and animal experiments should be performed to confirm these findings, especially the role of SCD1 in the effect of metformin on reducing hepatic TG accumulation. Second, though AMPK-SREBP-1c pathway may be associated with the effect of metformin on decreasing SCD1 expression, inhibition or activation of AMPK experiments should be done to further validate this hypothesis. And whether other factors are involved in the regulation of SCD1 by metformin is unclear. Third, the precise sequence of the SCD1 promoter regulated by metformin is uncertain.

## Conclusions

**Fig. 6:**
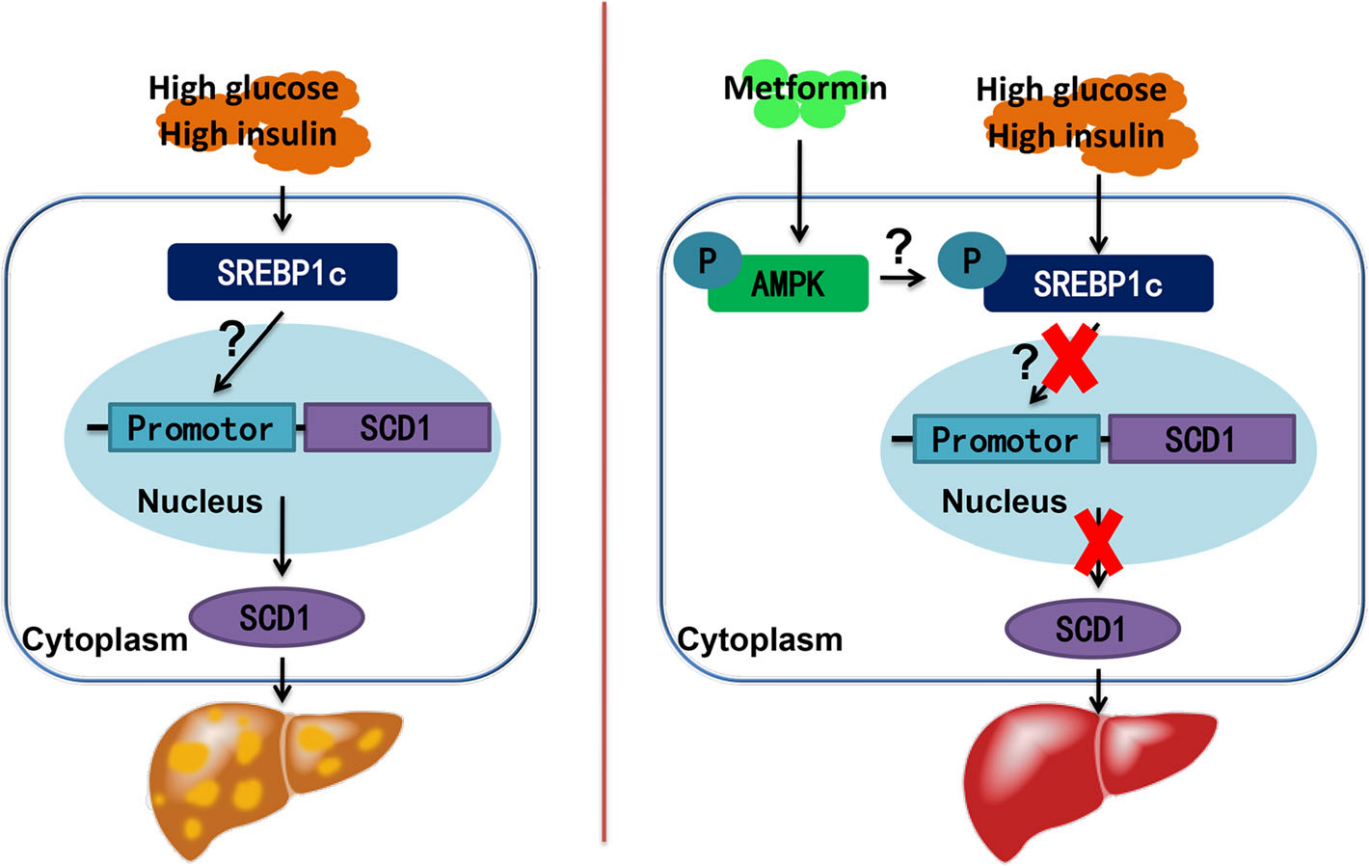
Proposed working model. High glucose and high insulin promote the expression of SCD1, which leads to increased TG accumulation in hepatocytes. Metformin inhibits the transcriptional activity of the SCD1 promoter, thus decreasing the expression of SCD1, which leads to reduced TG accumulation in hepatocytes. The AMPK-SREBP-1c pathway may be involved in the regulation of the transcriptional activity of the SCD1 promoter by metformin

In summary, our study shows that metformin attenuates TG accumulation in hepatocytes via decreasing SCD1 expression (Fig. 6). AMPK and SREBP-1c may be associated with the effect of metformin on decreasing SCD1 expression. Metformin is a potential effective agent for the treatment of NAFLD.

## Abbreviations

AMPK: Adenosine monophosphate-activated protein kinase
FAS: Fatty acid synthetase
HCC: Hepatocellular carcinoma
MOI: Multiplicity of infection
mRNA: Messenger RNA
MS: Metabolic syndrome
NAFLD: Nonalcoholic fatty liver disease
NASH: Nonalcoholic steatohepatitis
P/S: Penicillin/Streptomycin
PVDF: Polyvinylidenedifluoride
SCD1: Stearyl-coenzyme A desaturase 1
SEM: Standard error of the mean
shRNA: Small hairpin RNA
SREBP-1c: Sterol regulatory element-binding protein-1c
T2DM: Type 2 diabetes
TG: Triglyceride
